# Systematic screening and validation of reliable reference genes for qRT-PCR analysis in Okra (*Abelmoschus esculentus* L.)

**DOI:** 10.1038/s41598-022-16124-3

**Published:** 2022-07-28

**Authors:** Jing-Rong Zhang, Yuan-Yuan Feng, Ma-Jin Yang, Yu Xiao, Yu-Shan Liu, Yuan Yuan, Zhen Li, Yan Zhang, Ming Zhuo, Jun Zhang, Cai-Xia Li

**Affiliations:** 1 Biotechnology Research Institute, Sichuan Academy of Botanical Engineering, Neijiang, 641200 China; 2 College of Chemistry and Life Science, Chengdu Normal University, Chengdu, 611130 China; 3 Chengdu Institute of Biology, Chinese Academy of Sciences, Chengdu, 610041 China

**Keywords:** Molecular biology, Plant sciences

## Abstract

Quantitative real-time polymerase chain reaction (qRT-PCR) is a sensitive and widely used technique for quantifying gene expression levels, and its accuracy depends on the reference genes used for data normalization. To date, no reference gene has been reported in the nutritious and functional vegetable okra (*Abelmoschus esculentus* L.). Herein, 11 candidates of reference genes were selected and evaluated for their expression stability in okra in different tissues at different developmental stages by using three software algorithms (geNorm, NormFinder, BestKeeper) and a web-based tool (RefFinder). Among them, eukaryotic initiation factor 4 alpha (*eIF4A*) and protein phosphatase 2A (*PP2A*) showed the highest stability, while *TUA5* had the lowest stability. The combined usage of these two most stable reference genes was sufficient to normalize gene expression in okra. Then, the above results were further validated by normalizing the expression of the cellulose synthase gene *CesA4*. This work provides appropriate reference genes for transcript normalization in okra, which will facilitate subsequent functional gene research on this vegetable crop.

## Introduction

Okra (*Abelmoschus esculentus* L.), belonging to the Malvaceae family, is a healthy and nutritious vegetable crop widely consumed around the world. Its immature pods are good sources of essential minerals, vitamins, amino acids and edible dietary fibers^1^. Moreover, okra flowers and immature pods are rich in flavonoids and polysaccharides, which exhibit excellent anticancer effects^2^ and strong antioxidant activities^3^. Noteworthy, the okra pods have the highest nutritional content about 7 days after fruit setting, and then the pods age quickly^4^. If the okra pods are not harvested in time, it might cause a huge loss of the nutritional and economic value. According to the previous reports^5^, the cellulose content of okra pods increases greatly during their aging process. Currently, the molecular mechanism underlying the rapid aging of okra pods remains unclear. Thus, analyzing the expression patterns of key genes in the early developmental stage of okra pods will facilitate our understanding of the regulatory mechanisms regarding pod aging. To ensure the accuracy of gene expression analysis, appropriate reference genes and techniques need to be selected.

Quantitative reverse transcription-polymerase chain reaction (qRT-PCR) is now widely used as the gold standard for accurate and rapid measurement of gene expression^6^. However, its accuracy is greatly influenced by the expression stability of the reference genes used for data normalization to account for the differences in PCR efficiency and variation in sample content between reactions^7,8^. The optimal reference gene should remain relatively constant and display minimal variation across tissue types, developmental stages, and experimental conditions. Traditionally, housekeeping genes are usually used as the reference genes, but validation is poor due to their tendency to be constitutively expressed in various tissues^9^. In fact, even the most commonly used housekeeping genes may vary significantly in their stability across different species and tissues, or under different developmental stages and experimental conditions^10,11^. Therefore, screening appropriate reference genes for a given experimental condition and sample material is a prerequisite for gene expression analysis.

Recently, numerous stable reference genes have been validated for quantitative expression analyses in different plant species, such aspea^7^, tobacco^8^, soybean^12^, Siberian wild rye^13^, and goosegrass^14^. However, the okra reference gene has not yet been identified and validated, which greatly hinders the analysis of functional genes and molecular basis research, especially the rapid aging mechanism of okra pods. Therefore, it is necessary to evaluate the stability of candidate reference genes in different tissues and at different development stages in *A*. *esculentus*.

Herein, we aim to identify reliable reference genes for qRT-PCR data normalization in okra. Eleven genes includingactin 2 (*ACT2*), protein phosphatase 2A (*PP2A*), polyubiquitin 10 (*UBQ10*), 18S ribosomal RNA protein (*18SrRNA*), eukaryotic initiation factor 4 alpha (*eIF4A*), Low expression of osmotically responsive genes 1 (*Los1*), tubulin alpha 5 (*TUA5*), heterogeneous nuclear ribonucleoprotein (*hnRNP*), elongation factor 1-alpha (*EF1-α*), SAND family protein (*SAND*), and yellow leaf specific 8 (*YLS8*) were selected as candidate reference genes based on RNA-seq data from our lab. Their expression stabilities in the roots, stems, leaves, flowers and pods were systematically evaluated using geNorm^15^, NormFinder^16^, BestKeeper programs^17^ and a web tool RefFinder (http://blooge.cn/RefFinder). In addition, a targeted gene, involved in cellulose synthesis, namely *AeCesA4*, was used to validate the above reference genes.

## Results

### Verification of primer specificity and Cq values of candidate reference genes

**Figure 1:**
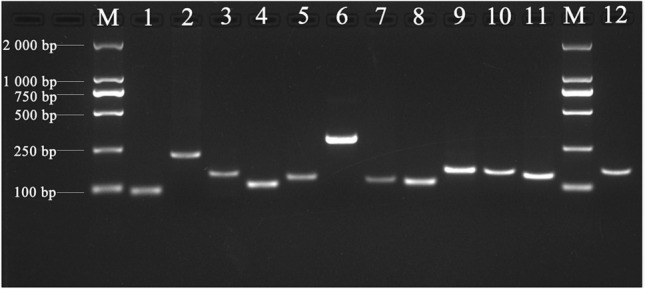
The PCR amplification specificities of 11 candidate reference genes and one targeted gene detected by agarose gel electrophoresis. M: DNA marker; 1: EF-1α, 2: YLS8, 3: PP2A, 4: TUA5, 5: SAND, 6: 18S rRNA, 7: hnRNP, 8: eIF4A, 9: UBQ10, 10: LOS1, 11: ACT2, 12: CesA4.

A total of 11 candidate reference genes were selected for qRT-PCR normalization (Table 1). To check the specificity of primers used in PCR reactions, agarose gel electrophoresis (1.8% w/v) and melting curve analyses were performed. The results showed that a single band was obtained in each lane, yielding a single amplification product with expected size (Fig. 1). Meanwhile, the melting curve analysis showed that all of the primers amplified single major peaks (Fig. S1). These results indicate that all the primers pairs are highly specific.

**Table 1:** Details of primers used in this study.

Gene symbol	Gene name	Primer sequence (5′–3′)	Product (bp)	E (%)	R^2^
*eIF4A*	Eukaryotic initiation factor 4 alpha	F: ATGCATATGGTTTTGAGAAGCC	115	101.4	0.993
		R: AAAGTTGCAGTCTTCCCAGTTC			
*ACT2*	Actin 2	F: ACACTGTGCCAATCTATGAAG	145	96.5	0.997
		R: ACAATTTCCCGCTCAGCAGTG			
*18S rRNA*	18S ribosomal RNA protein	F: ATAACTCGACGGATCGCACG	304	102.2	0.997
		R: CTTGCCCTCCAATGGATCCT			
*UBQ10*	Polyubiquitin 10	F: ATAATACCACCACGAAGACGG	158	86	0.999
		R: GTCAAGCAAAAGATTCAAGACAAGG			
*PP2A*	Protein phosphatase 2A	F: GACATCATGTCCATGTTTGATG	149	88.7	0.999
		R: TGTGAGAAATTAACAATGACAG			
*hnRNP*	Heterogeneous nuclear ribonucleoprotein	F: CATGCAACCAATAAGTCTCGTG	124	92.2	0.997
		R: CTTTCTTGATCTCCACCTGGGT			
*SAND*	SAND family protein	F: CATACACTTGTCTTCCTCT	129	88.6	0.999
		R: GCACCAACAAGACTGATAA			
*EF1-α*	Elongation factor 1-alpha	F: TTGCCGTCAAATTTGCTGAACT	90	104.2	0.995
		R: CTCCGTTCTTCAAGAACTTAGG			
*TUA5*	Tubulin alpha 5	F: GGGAAGTACATGGCATGCTGCC	108	84.9	0.997
		R: GTCAACAAACTGCACAGTCCTC			
*YLS8*	Yellow leaf specific 8	F: CGACTGGGATGAGACTTGT	234	88.6	1.000
		R: CTGTTTGTCCTTGAGAGCC			
*LOS1*	Low expression of osmotically responsive genes 1	F: AGCAGGAAACTGTTGAGGA	124	99.3	0.999
		R: CAGCAACACGAACAACAGGA			
*CesA4*	Cellulose synthase A4	F: TCTGTGATCTGCGAAGTCT	163	92.2	0.999
		R: CGGTACTTACGAACACATC

**Figure 2:**
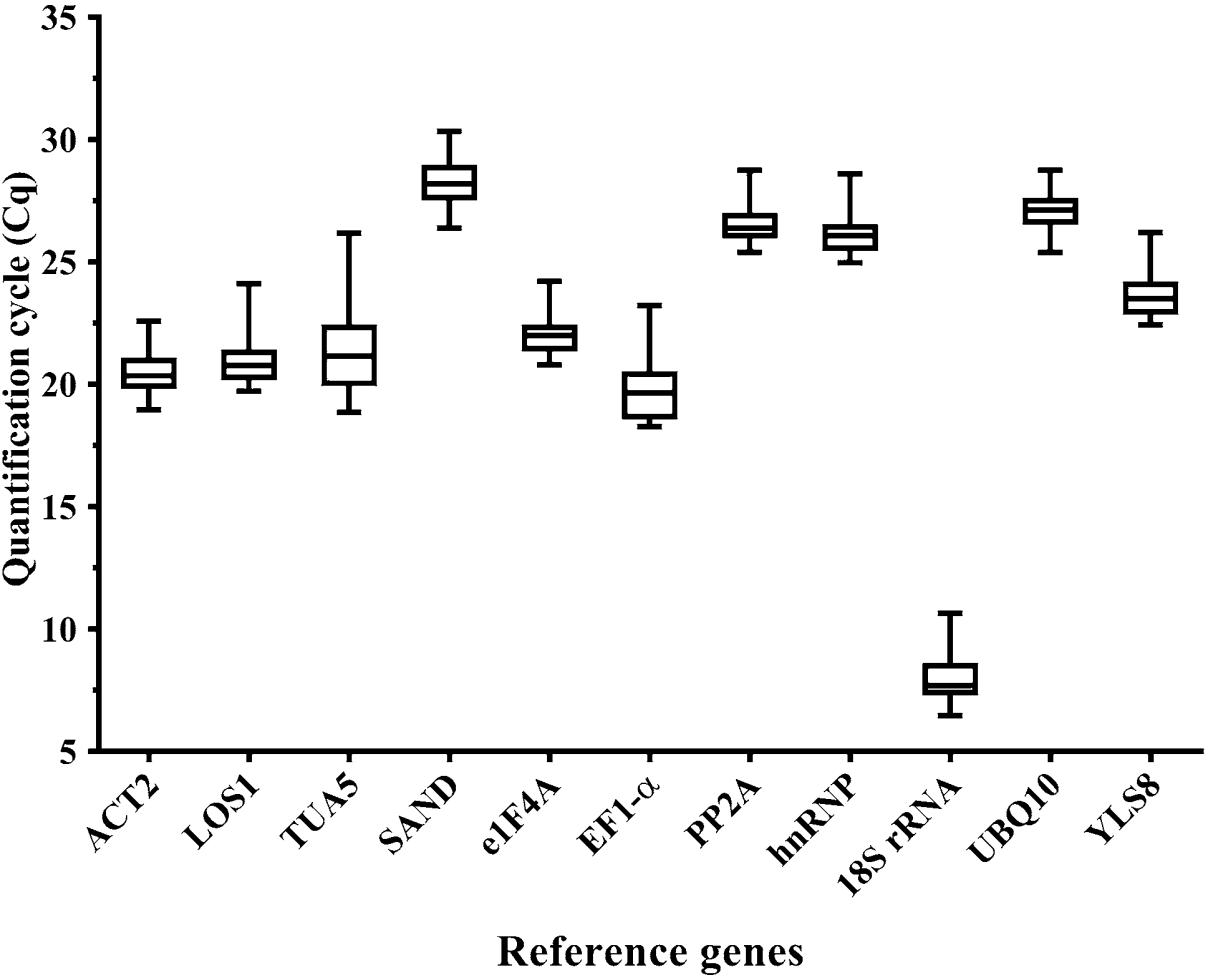
Cq values of 11 reference genes across all samples. The whiskers of the boxes are the maximum and minimum Ct values, and the horizontal lines inside the boxes represent the median of each reference gene.

The expression levels of candidate genes were detected in all samples according to the quantification cycle values (Cq values) obtained by qRT-PCR, and the mean Cq values of these candidates were between 7.94 (*18S rRNA*) and 28.23 (*SAND*), showing a wide range of expression levels (Fig. 2). Since gene expression levels are negatively correlated to Cq values, *18S rRNA* was the most expressed gene with the lowest mean Cq value, while *SAND* was the least abundant gene with the highest mean Cq value among the 11 candidate reference genes.

### Expressing stability analysis

Expression stability of 11 candidate genes was analyzed by geNorm, NormFinder and BestKeeper independently and the ranking of their stability was obtained separately. Then we got a comprehensive ranking using the web tool, RefFinder that integrates aforementioned three algorithms plus the Delta CT method.

### geNorm analysis

Based on the geNorm analysis, the mean (M) values of all candidates ranging from 0.155 to 0.928 (Table 2), were lower than the cutoff value of 1.5 in all samples, indicating that all of the candidate genes were relatively stable in okra. In pod and leaf groups, *eIF4A* and *LOS1* were found to be most stable, while *18S rRNA* and *TUA5* exhibited low stability and were ranked as the least stable ones in leaf and pod group, respectively. For different tissues of young seedlings, *LOS1* and *PP2A* with the lowest value of 0.155 showed the best stability, whereas *UBQ10* displayed the worst stability. For different organs in the fruiting period, the two most stable genes were *eIF4A* and *PP2A*, while *TUA5* with the highest value of 0.979 was the most unstable. For the all samples, *YLS8* and *PP2A* were the most stable genes, followed by *hnRNP*, but *TUA5* was the least stable one. Among all the groups, *eIF4A* and *LOS1* showed higher stability, whereas *TUA5* had the lowest stability in most groups.

**Table 2:** Expression stability analysis of reference genes assayed by geNorm, NormFinder, BestKeeper, and RefFinder.

Group	Rank	geNorm		NormFinder		BestKeeper			RefFinder	
		Gene	Stabitily	Gene	Stabitily	Gene	SD	CV	Gene	Stabitily
Total	1	*YLS8*	0.425	*eIF4A*	0.12	*UBQ10*	0.53	1.96	*eIF4A*	1.86
	2	*PP2A*	0.425	*ACT2*	0.21	*hnRNP*	0.58	2.21	*PP2A*	2.63
	3	*hnRNP*	0.447	*LOS1*	0.239	*eIF4A*	0.62	2.79	*YLS8*	3.6
	4	*eIF4A*	0.466	*PP2A*	0.252	*YLS8*	0.64	2.73	*hnRNP*	3.66
	5	*LOS1*	0.494	*SAND*	0.357	*18S rRNA*	0.66	8.31	*ACT2*	3.98
	6	*ACT2*	0.526	*hnRNP*	0.365	*PP2A*	0.67	2.53	*LOS1*	4.68
	7	*SAND*	0.584	*YLS8*	0.379	*ACT2*	0.7	3.4	*UBQ10*	5.62
	8	*18S rRNA*	0.636	*18S rRNA*	0.464	*LOS1*	0.76	3.62	*SAND*	6.85
	9	*UBQ10*	0.691	*EF1-α*	0.528	*SAND*	0.78	2.76	*18S rRNA*	7.11
	10	*EF1-α*	0.74	*UBQ10*	0.618	*EF1-α*	0.98	4.98	*EF1 a*	9.24
	11	*TUA5*	0.844	*TUA5*	0.841	*TUA5*	1.4	6.52	*TUA5*	11
Pods	1	*eIF4A*	0.219	*eIF4A*	0.093	*EF1-α*	0.41	2.16	*eIF4A*	1.5
	2	*LOS1*	0.219	*LOS1*	0.098	*UBQ10*	0.45	1.63	*LOS1*	2
	3	*PP2A*	0.238	*PP2A*	0.197	*ACT2*	0.61	3.01	*PP2A*	3.83
	4	*hnRNP*	0.309	*SAND*	0.245	*eIF4A*	0.71	3.23	*ACT2*	4.61
	5	*YLS8*	0.345	*ACT2*	0.264	*LOS1*	0.71	3.41	*SAND*	5.09
	6	*ACT2*	0.407	*YLS8*	0.284	*SAND*	0.73	2.67	*EF1-α*	5.62
	7	*SAND*	0.448	*18S rRNA*	0.329	*18S rRNA*	0.75	9.77	*UBQ10*	5.83
	8	*UBQ10*	0.483	*hnRNP*	0.337	*PP2A*	0.76	2.86	*YLS8*	6.51
	9	*18S rRNA*	0.52	*UBQ10*	0.36	*hnRNP*	0.95	3.63	*hnRNP*	6.7
	10	*EF1-α*	0.55	*EF1-α*	0.426	*YLS8*	1	4.19	*18S rRNA*	7.94
	11	*TUA5*	0.658	*TUA5*	0.747	*TUA5*	1.58	7.69	*TUA5*	11
Leaves	1	*eIF4A*	0.148	*eIF4A*	0.032	*eIF4A*	0.24	1.07	*eIF4A*	1
	2	*LOS1*	0.148	*SAND*	0.046	*ACT2*	0.26	1.23	*LOS1*	2.82
	3	*EF1-α*	0.167	*LOS1*	0.057	*SAND*	0.26	0.9	*SAND*	2.83
	4	*SAND*	0.174	*EF1-α*	0.057	*hnRNP*	0.26	0.99	*ACT2*	3.98
	5	*ACT2*	0.196	*ACT2*	0.127	*YLS8*	0.27	1.16	*EF1-α*	4.12
	6	*YLS8*	0.212	*YLS8*	0.128	*EF1-α*	0.31	1.54	*hnRNP*	5.66
	7	*hnRNP*	0.228	*hnRNP*	0.158	*LOS1*	0.33	1.56	*YLS8*	5.73
	8	*PP2A*	0.254	*PP2A*	0.185	*PP2A*	0.36	1.37	*PP2A*	8
	9	*UBQ10*	0.311	*UBQ10*	0.397	*UBQ10*	0.41	1.5	*UBQ10*	9
	10	*TUA5*	0.378	*TUA5*	0.41	*TUA5*	0.49	2.25	*TUA5*	10
	11	*18S rRNA*	0.462	*18S rRNA*	0.554	*18S rRNA*	0.71	8.65	*18S rRNA*	11
Young seedling	1	*LOS1*	0.155	*eIF4A*	0.04	*18S rRNA*	0.24	3.17	*LOS1*	1.68
	2	*PP2A*	0.155	*LOS1*	0.139	*LOS1*	0.31	1.52	*eIF4A*	1.86
	3	*eIF4A*	0.246	*PP2A*	0.207	*PP2A*	0.32	1.23	*PP2A*	2.28
	4	*hnRNP*	0.284	*SAND*	0.209	*eIF4A*	0.32	1.49	*18S rRNA*	4.3
	5	*SAND*	0.349	*hnRNP*	0.213	*hnRNP*	0.37	1.42	*hnRNP*	4.47
	6	*YLS8*	0.384	*ACT2*	0.249	*SAND*	0.4	1.42	*SAND*	5.14
	7	*18S rRNA*	0.411	*EF1-α*	0.291	*TUA5*	0.4	1.89	*ACT2*	6.93
	8	*ACT2*	0.443	*18S rRNA*	0.292	*ACT2*	0.45	2.24	*YLS8*	8.13
	9	*EF1-α*	0.47	*YLS8*	0.337	*YLS8*	0.46	2.01	*EF1-α*	8.71
	10	*TUA5*	0.509	*TUA5*	0.43	*EF1-α*	0.59	3.06	*TUA5*	8.8
	11	*UBQ10*	0.555	*UBQ10*	0.469	*UBQ10*	0.85	3.18	*UBQ10*	11
Fruiting period	1	*eIF4A*	0.264	*eIF4A*	0.091	*UBQ10*	0.39	1.42	*eIF4A*	1.50
	2	*PP2A*	0.264	*PP2A*	0.128	*hnRNP*	0.79	2.99	*hnRNP*	5.66
	3	*ACT2*	0.346	*ACT2*	0.196	*YLS8*	0.84	3.49	*PP2A*	2.30
	4	*LOS1*	0.424	*LOS1*	0.240	*eIF4A*	0.91	4.06	*YLS8*	3.35
	5	*SAND*	0.471	*SAND*	0.302	*PP2A*	0.91	3.36	*ACT2*	3.57
	6	*YLS8*	0.564	*YLS8*	0.416	*ACT2*	0.91	4.40	*LOS1*	4.90
	7	*hnRNP*	0.602	*hnRNP*	0.452	*18S rRNA*	0.76	9.52	*UBQ10*	5.48
	8	*18S rRNA*	0.653	*18S rRNA*	0.474	*LOS1*	1.11	5.18	*SAND*	5.62
	9	*UBQ10*	0.737	*EF1-α*	0.692	*SAND*	0.94	3.83	*18S rRNA*	5.66
	10	*EF1-α*	0.820	*UBQ10*	0.780	*EF1-α*	1.30	6.54	*EF1-α*	9.49
	11	*TUA5*	0.980	*TUA5*	1.115	*TUA5*	2.19	10.18	*TUA5*	11.00

**Figure 3:**
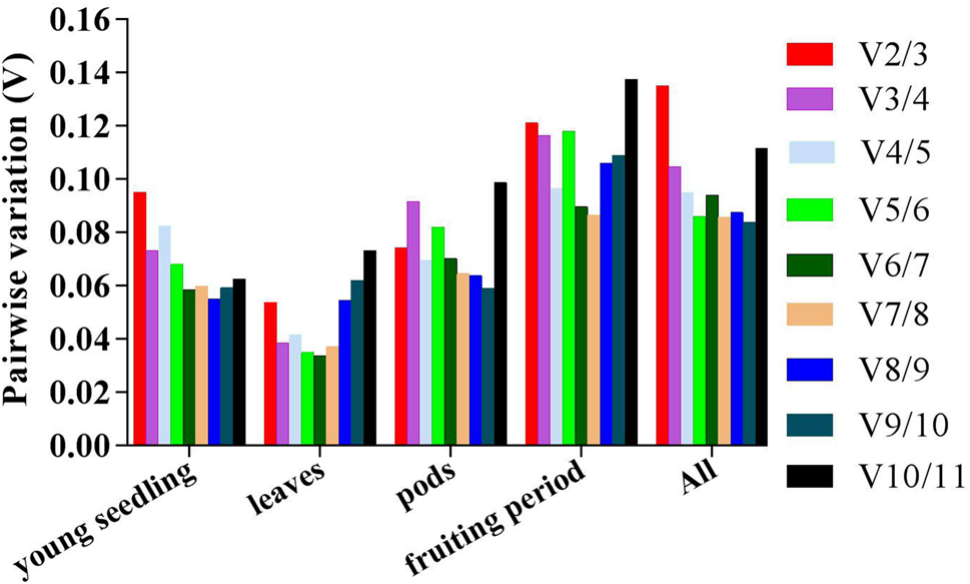
Optimal number of reference genes required for qRT-PCR data normalization by determining the pairwise variation (V).

The geNorm program was also used to analyze pairwise variation values of Vn/Vn + 1 for the assessment of the minimal number of reference genes required for normalization. For the total samples, a minor variation was found between V2/3 (0.135) and V3/4 (0.105), suggesting that the two reference genes (*YLS8* and *PP2A*) would be suitable for normalization. For the other groups, both V2/3 and V3/4 values were less than 0.15 (Fig. 3), indicating that the use of the top two reference genes was sufficient for normalization in qRT-PCR.

### NormFinder analysis

Expression stability values, intra- and inter-group variances of candidate genes in groups 1 and 2, groups 1 and 3, and groups 1 and 4 analyzed by NormFinder are shown in Table 2 and Table S1. Among all the groups, *eIF4A* got the top rank, which was somewhat different from geNorm results. For example, according to the geNorm analysis, *PP2A* showed the highest stability in seedling group, whereas its stability ranked third in the NormFinder analysis. Nevertheless, the most unstable reference genes in all groups were consistent with the results of the geNorm analysis. In general, *eIF4A* exhibited the best expression stability, while *TUA5* and *UBQ10* performed poorly across all groups.

### BestKeeper analysis

BestKeeper assesses expression stability by measuring the standard deviation (SD) and coefficient of variance (CV). The more stable reference gene possessed the lower SD (i.e., usually < 1) value. For different tissues in the fruiting period and total samples group, *UBQ10* and *hnRNP* were the most stable reference genes, whereas *TUA5* with a SD value > 1 was considered as an unstable gene. In the leaf group, all reference genes had lower SD values (SD ≤ 0.71), and *eIF4A* and *ACT2* were considered as the most suitable reference genes, and *18S rRNA* obtained the lowest stability. For pod samples, *EF1-α* and *UBQ10* were the optimal reference genes, while *TUA5* was unacceptable owing a higher SD value of 1.58. For different tissues in the seedling group, *18S rRNA* and *LOS1* were placed as the best reference genes, while *UBQ10* as the worst one.

### RefFinder analysis

RefFinder, an online tool for expression stability of reference genes, was used to calculate and recommended comprehensive ranking of 11 candidates based on the three previously described algorithms and delta-Ct^18^ (Table 2). The comprehensive rankings from RefFinder showed that *eIF4A* and *PP2A* had the highest stability, while *EF1-α* and *TUA5* had the least stability across all samples. For different tissues in the fruiting period, *eIF4A* and *hnRNP* were the two most stable reference genes. For pods and leaves at different developmental stages and different tissues of the young seedlings, the top two genes were *eIF4A* and *LOS1*, while *UBQ10*, *TUA5* and *18S rRNA* was ranked as the most unstable gene in the seedling stage, pods and leave groups, respectively. Taken together, *eIF4A* was defined as the most stably expressed gene, while *TUA5* was the least stable in most groups.

### Validation of the stability of reference genes

To test and verify the reliability of the screened reference genes, a target gene needs to be selected for qRT-PCR amplification. The relative expression pattern of gene *CesA4*, which encodes an enzyme essential for cellulose biosynthesis in plants, was tested in pods at different developmental stages, as well as in different tissue samples. And its relative expression levels were normalized using two most stable genes (*eIF4A* and *PP2A* for different tissues, *eIF4A* and *LOS1* for pods), and the least stable reference gene (*TUA5* and *EF1-α* for different tissues, *TUA5* and *18S rRNA* for pods), as well as one moderately stable reference gene *ACT2* based on the results of RefFinder.

**Figure 4:**
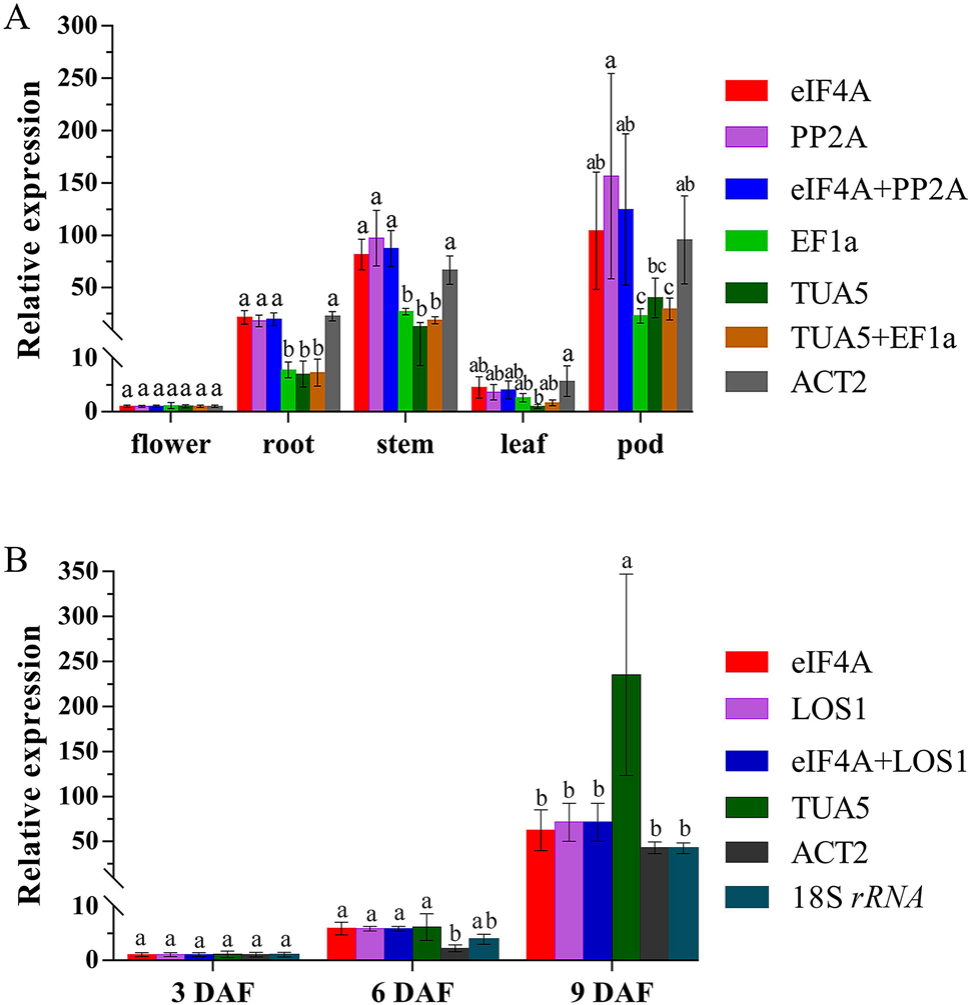
Validation of the reference genes by the relative expression of target gene AeCesA4 in different tissues (**A**) and pods at different developmental stages (**B**) The most two stable reference genes (eIF4A and PP2A for different tissues, eIF4A and LOS1 for pods), the least stable reference genes (TUA5 and EF1-α for different tissues, TUA5 and 18S rRNA for pods), as well as one moderately stable reference geneACT2 recommended by RefFinder were selected as normalization factors. Data represent the mean ± standard error of three independent replicates, different superscript letter on the vertical bars indicate significantly different at p < 0.05.

The qRT-PCR analysis showed that *AeCesA4* expression was the highest in 9 DAF pods, followed by stems at the young seedling phase, but lower in flowers and leaves (Fig. 4A). In different tissues, the expression patterns of *AeCesA4* were similar when normalized using *eIF4A* and *PP2A* alone or in combination, but the relative expression levels of *AeCesA4* decreased significantly in roots, stems, and 9 DAF pods (*p* < 0.05), when normalized with *EF1-α* and *TUA5* (Fig. 4A). On the other hand, when *TUA5* was used as an internal gene, the relative expression level of *AeCesA4* in 9 DAF pods was much higher than those with stable genes (*eIF4A* and *LOS1*) (*p* < 0.05) (Fig. 4B). When normalized by *ACT2* and *18S rRNA* independently, however, the relative expression level of *AeCesA4* was lower compared to normalization by the optimal genes (Fig. 4B). In the pods group, data normalization using the most widely used reference genes *ACT2*, the relative expression level of *AeCesA4* in 6 DAF pod samples were significantly underestimated (*p* < 0.05) (Fig. 4B), thus highlighting the importance of selecting suitable reference genes.

## Discussion

Presently, qRT-PCR is regarded as the best choice for accurately analyzing gene expression levels in different samples. However, due to its high sensitivity, this technique is highly subjected to manipulation level and samples’ variations. When inappropriate reference genes are used for normalization analysis, these changes can severely affect results. Therefore, the selection of suitable reference genes is crucial to ensure the accuracy of qRT-PCR. However, systematic screening of reference genes of okra (*A. esculentus*) has not been reported.

Okra is an important vegetable which is popular all over the world. Despite of its high nutritional and medicinal effects, little attention has been paid to its molecular function and gene expression. Until now, its genome has not been sequenced, and little is known about the molecular mechanisms of the pod growth and development. Also, a set of reliable reference genes for qRT-PCR assay is still lacking. Fortunately, RNA sequencing (RNA-seq) is now a powerful approach for transcriptome analysis of differential gene expression. And it provides a resource for the identification of reference genes in non-model plants without genome information. Here, we used RNA-seq approach to identify the suitable reference genes for accurate normalization of the transcript levels by qRT-PCR analyses in okra.

In the present study, 11 candidate internal reference genes (*ACT2*, *LOS1*, *TUA5*, *hnRNP*, *SAND*, *EF1-α*, *eIF4A*, *YLS8*, *PP2A*, *UBQ10*, *18S rRNA*) were identified from our transcriptome data. The three most extensively used software packages (geNorm^15^, NormFinder^16^, BestKeeper^17^) and one web tool RefFinder were used to assess the expression stability of the candidate reference genes. Four programs showed a few differences in results, for example, according to the NormFinder evaluation, *eIF4A* and *ACT2* were the most two stable reference genes in all of the samples examined, whereas their stability rankings were fourth and sixth in the geNorm analysis, respectively. However, results from BestKeeper analyses showed that *UBQ10* and *hnRNP* were the most suitable reference genes in all the tested samples. Analysis using geNorm and Normfinder resulted in different orders of most stable genes but the least stable reference genes were the same ones. In general, stability ranking of reference genes generated by BestKeeper was quite different from those of the other two algorithms, similar to the results of previous reports^19,20^. It was difficult to determine the stable reference genes in *A. esculentus* using only one algorithm. Therefore, we used RefFinder which integrates the other computational algorithm to counteract bias and to obtain a comprehensive ranking of gene expression stability. Unexpectedly, *eIF4A* was defined as the most stably expressed gene in all tissues and specific tissue groups examined in this study. Moreover, previous studies have proved that *eIF4A* was suitable for normalization in gene expression studies in *Avena sativa* L. and *Eleusineindica*^14,21^. Following *eIF4A*, *PP2A* also displayed particularly excellent stability among all samples and it has been reported as a stably expressed gene in other species^8,13,22,23^. For these reasons, *eIF4A* and *PP2A* recommended by the above-mentioned software could be accepted as reference genes in this work. In contrast, *TUA5* was the least recommended reference gene in most groups of this study, while *TUA5* exhibits highly stable expression across development in soybean and in different tissues of *Suaeda glauca*^12,22^.

While for accurate normalization of qRT-PCR results, a single reference gene usually cannot meet the requirements^15,24^. The optimal number and choice of reference genes must be determined experimentally and methodically^25^. In the current study, the pairwise variation parameters from geNorm calculated indicated that a combination of two top stable reference genes may be a better option for gene expression normalization in all cases. Based on the comprehensive ranking of RefFinder, the combination of *eIF4A* and *PP2A* was the most stable reference gene set for all samples in our research. The best reference gene set for developing pods, young seedling samples and leaf, was *eIF4A* plus *LOS1*, and the optimal reference gene set for different tissues in the fruiting period was *eIF4A* plus *hnRNP*.

Cellulose, the main component of plant cell walls, plays a vital role in the growth and development of plants. The gene *cellulose synthase A* (*CesA*), encoding cellulose synthases, is responsible for cellulose biosynthesis in plant cell walls. Currently, *CesA* genes have been extensively studied in model plants such as rice and *Arabidopsis*^26,27^. Nevertheless, the regulatory mechanisms of *CesA* expression are not well investigated in *A. esculentus*. To confirm the expression stability of reference genes in the current study, the relative expression patterns of *AeCesA4*, one of secondary cell wall-associated cellulose synthase genes, was analyzed by qRT-PCR.

The results showed large differences in the quantification of *AeCesA4* expression level when normalized using the best reference gene compared to the least stable one. For instance, when the least stable reference gene *TUA5* was used, *AeCesA4* expression levels were underestimated significantly in roots, stems and leaves (*p* < 0.05) (Fig. 4A), whereas the opposite results were displayed in 9 DAF pods (*p* < 0.05) (Fig. 4B). Actually, the mean Cq values of *TUA5* ranging from 19.33 (3 DAF pods) to 22.96 (9 DAF pods) in pod group displayed relatively high variation around 3.33 cycles, indicating its expression levels decreased dramatically in later stages of pod development. Therefore, we are not surprised that when the least stable gene *TUA5* was used for normalization, the expression level of *AeCesA4* significantly increased compared to that of *eIF4A*, *LOS1*, or the combination of *eIF4A* + *LOS1* in 9 DAF pods (Fig. 4B).

Previous studies published on qRT-PCR in okra, usually *ACT* were used as a single internal control for qRT-PCR analysis^28,29^, and their stability has not yet been reported. However, in the present experiments, *ACT2* was ranked as moderately stable candidate reference gene according to RefFinder analysis. When *ACT2* was used for normalization analysis, the expression of *AeCesA4* in 6 DAF pods was significantly changed compared with the stable reference genes, was very similar to that in *Arabidopsis pumila*^30^ (*p* < 0.05) (Fig. 4B). Another most commonly used reference gene, *18S rRNA*, whose transcript abundance in okra was too high with Ct values less than 11, thus may affect the quantitative accuracy of the target gene. Similarly, misinterpretation was also observed in previous study^31^. Therefore, *18S rRNA* should be excluded according to the selected reference genes criteria proposed by Beillard^32^. Hence, *ACT2* and *18S rRNA*, although the most commonly used, are not the appropriate reference genes for okra. Our results indicated that the expression stability of commonly used reference gene may vary significantly across different tissues and different development stages, and further proved the importance of validating the normalizing reference genes before conducting gene expression analysis.

Importantly, the expression patterns of *AeCesA4* gene in okra’s tissues exhibited the higher expression in fast-growing pods and stems than other tissues. Similar expression patterns in *Miscanthus* × *giganteus* have been reported^33^; thus, it is consistent with its biological role of *CesAs* responsible for the secondary cell wall synthesis. Validation of gene expression revealed that *AeCesA4* showed similar expression patterns when using the single most stable reference gene and the most stable reference genes combinations, whereas the expression levels were significantly different when normalized using the most unstable reference genes, suggesting that the identified reference genes are reliable.

Note that the combinations of multiple reference genes can be expected to be more precise than a single one^9,24^. Based on validation results of target gene *AeCesA4* expression among different tissues, although its expression patterns is almost the same when normalized with the optimal gene or the combination of the two top stable reference genes, we recommend that using the appropriate combinations of two genes for more accurate and reliable qRT-PCR results for okra.

## Conclusions

This is the first systematic study to validate a set of candidate reference genes for normalization of qRT-PCR data in okra using four algorithms. Different sets of reference genes were recommended to normalize gene expression data in different tissues and at different development stages. For the total samples group, the combination of *eIF4A* and *PP2A* was the most stable reference gene set. The best reference gene set for developing pods, seedling samples and leaves of different developmental stages was *eIF4A* + *LOS1*, and the optimal reference gene set for different tissues in the fruiting period was *eIF4A* + *hnRNP*. Additionally, the expression patterns of target gene *AeCesA4* was determined to confirm the reliability of the selected reference genes. Our findings will benefit the qRT-PCR-based studies of gene expression in okra.

## Materials and methods

### Plant materials

The okra variety ‘lüwuxing’ used in this study was formally identified by associate research fellow Wei-Xia Liu (Chinese Academy of Tropical Agricultural Sciences). The voucher specimen of *A*. *esculentus* has been deposited in Shanghai Natural History Museum (Branch of Shanghai Science & Technology Museum) (Herbarium ID 92068). All experimental procedures were in accordance with local and national regulations. Okra seeds were placed on filter paper in 150 mm petri dishes, and an appropriate amount of distilled water was added. The dishes were placed in an incubator at 30°C for about 30 h. The sprouting seeds were sown in trays containing a mixture of peat soil, vermiculite, and perlite (1:1:1, v/v/v) and grown in a greenhouse under natural conditions for 1 month. The seedlings were moved outside of green house for hardening off in the open air for 1 week and then transplanted into the field (103°49′30.6″ E; 30°48′52.25″ N), Chengdu, China. Tissue samples of roots, stems, and young leaves were collected from 5-week-old seedlings. Mature and senescent leaves, before-blooming and full-blooming flowers, and pods (3, 6 and 9 days after flowering (DAF)) were harvested from the fruiting stage of plants. All of the samples were collected from three plants, and frozen in liquid nitrogen, then stored at − 80°C until RNA extraction. To analyze the stability of candidate reference genes, samples were divided into four groups. Group one, young seedlings, contains three different tissues (roots, stems, and leaves) from young seedlings. Group two, leaves, contains three developmental stages of leaves (young, mature, and senescent leaves). Group three, Pod, contains three developmental stages of pods (3-, 6-, and 9-day-old ones). Group four, fruiting period, contains five developmental stages of fruit (buds, flowers, 3-, 6-, and 9-day-old pods).

### RNA extraction and cDNA synthesis

Total RNA was extracted using Plant Total RNA Isolation Kit Plus (FOREGENE, Chengdu, China) according to the manufacturer’s instructions. The concentration and purity of extracted RNA was measured by a NanoDrop2000 (Thermo Scientific, USA), and its integrity was evaluated by 1.8% (w/v) agarose gel electrophoresis. Only the RNA absorbing ratio of 1.8–2.0 at OD260 nm/OD280 nm were used for further cDNA synthesis with the Prime Script^™^ RT reagent Kit with gDNA Eraser (TaKaRa, RR047A). The synthesized cDNAs were verified by RT-PCR and diluted tenfold for qRT-PCR analyses.

### Reference genes selection and primer design

Eleven candidate reference genes, including six traditional reference genes (*ACT2*, *18SrRNA*, *UBQ10*, *EF1-α*, *LOS1*, and *TUA5*) and the other five genes (*eIF4A*, *PP2A*, *SAND*, *YLS8*, and *hnRNP*) were selected as candidate genes based on their FPKM and fold change values from the transcriptome sequencing data of ‘lüwuxing’ pods (unpublished data). The primers for qRT-PCR were designed by the Primer Premier version 5.0^12^.

### qRT-PCR

The qRT-PCR was carried out a BIO-RAD CFX96 quantitative PCR instrument (BIO-RAD, Hercules, CA, USA). A final 10 μL reaction mixture was containing tenfold diluted cDNA 2 μL, 2 × SYBR Premix Ex TaqII(TilRNaseH Plus, TaKaRa) 5 μL, 0.15 μL each of 10 μM Forward and Reverse Primers, and 2.7 μL DNase/RNase free water. The amplification procedure were 95°C for 30 s, followed by 40 cycles of 95°C for 5 s, 55°C for 30 s and 72°C for 30 s. The melting curve was analyzed to determine primer specificity. A standard curve was achieved for each gene by tenfold continuous dilution of the product of the first amplification reaction, and 10^−4^, 10^−5^, 10^−6^, 10^−7^, 10^−8^ of which are used as the template. Amplification efficiency (E) was calculated based on the slope of the standard curve according to the formula: E = 10^–1/slope^ − 1. All qRT-PCR assays were performed in triplicate.

### Evaluation of reference genes

The raw data of qRT-PCR were obtained by the CFX equipment software, the average Cq values were used for further analyses. The expression stability of candidate reference genes was evaluated with three algorithms namely geNorm v3.5 (https://genorm.cmgg.be/)^15^, NormFinder v0.953 (https://moma.dk/normfinder-software)^16^, and BestKeeper v1.0 (https://www.gene-quantification.de/bestkeeper.html)^17^, and then a comprehensive ranking was obtained by the RefFinder program (http://blooge.cn/RefFinder). The analysis methods of these programs were the same as those described in previous study^13^.

### Validation of reference genes

To verify the stability of reference genes, qRT-PCR was performed to detect the expression patterns of Cellulose synthase gene *AeCesA4* in different tissue samples (root, stem, leaf, flower and pod) and pods at different developmental stages. The relative expression level of *AeCesA4* was calculated by 2^−ΔΔct^ method^34^. One-way analysis of variance (ANOVA) test was applied to analyze significant differences among the reference genes using SPSS statistical software 19 (*p* < 0.05, Duncan’s multiple range tests)^30^.

## Supplementary Information

Supplementary Information.

## Data Availability

The raw data of transcriptome sequencing were submitted to NCBI Sequence Read Archive (SRA) with accession number Bioproject: PRJNA861710.
