# Supplementary material for: Systematic screening and validation of reliable reference genes for qRT-PCR analysis in Okra (Abelmoschus esculentus L.)
Source: Sci Rep. 2022 Jul 28;12:12913. doi: 10.1038/s41598-022-16124-3 (PMC9334609; doi:10.1038/s41598-022-16124-3)
Supplement: Supplementary file 1 — Supplementary Information. [file 41598_2022_16124_MOESM1_ESM.pdf]

Systematic screening and validation of reliable reference genes for qRT-PCR analysis in Okra (*Abelmoschus esculentus* L.)

Jing-Rong Zhang [1,+,*], Yuan-Yuan Feng[2,+], Ma-Jin Yang [1], Yu Xiao[3], Yu-Shan Liu [1],Yuan Yuan[2], Zhen Li [1], Yan Zhang [1], Ming Zhuo[1] , Jun Zhang [1],Cai-Xia Li[3,] *

[1] Biotechnology Research Institute, Sichuan Academy of Botanical Engineering, Neijiang 641200, China

[2] College of Chemistry and Life Science, Chengdu Normal University, Chengdu 611130, China

[3] Chengdu Institute of Biology, Chinese Academy of Sciences, Chengdu 610041, China

[*]Corresponding. zjr_sc@163.com;licx@cib.ac.cn

[+]These authors contributed equally to this work

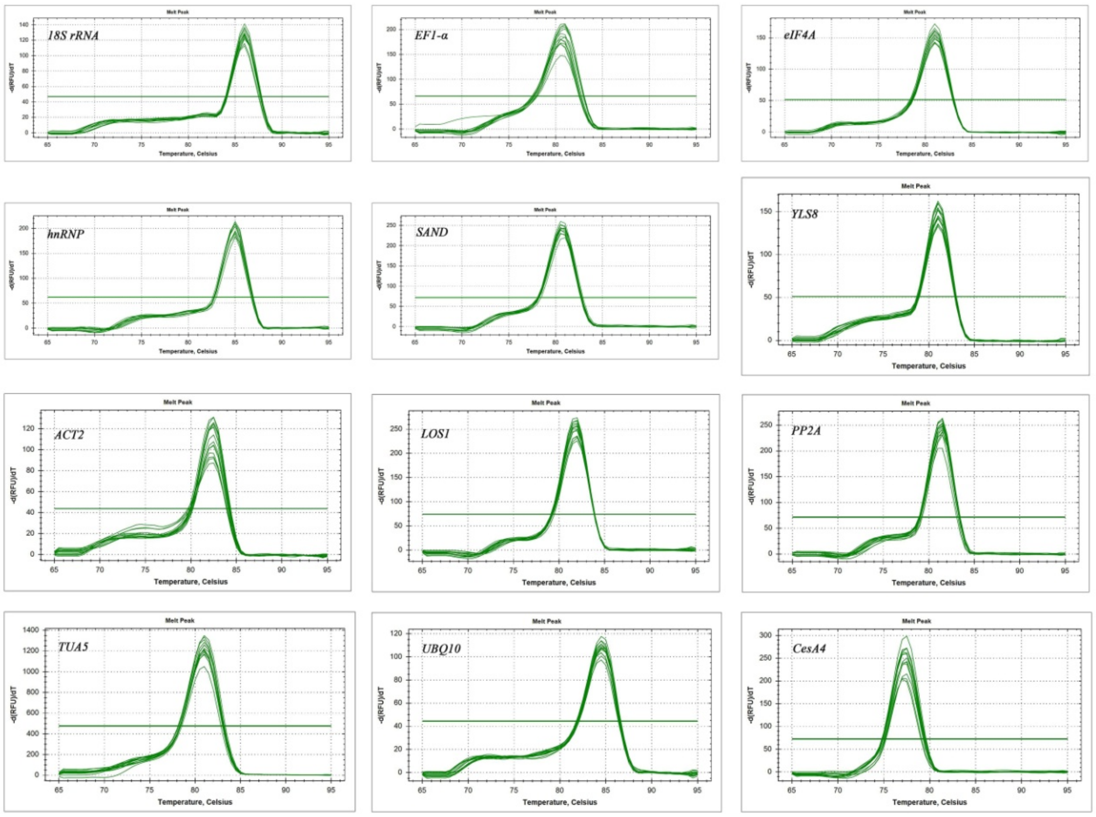

Figure. S1. The melting curves of 11 candidate reference genes and one target gene in *Abelmoschus esculentus*. The melting curves of qRT-PCR amplifications were obtained by the Bio-Rad CFX Manager software version 3.0 (http://www.bio-rad.com/cfxmanagersoftware).

candidate reference genes	Group 1 and group 2						Group 1 and group 3						Group 1 and group 4					
	Stability value	Intra-group variance			Inter-group variance		Stability value	Intra-group variance			Inter-group variance		Stability value	Intra-group variance			Inter-group variance	
		Group 1	Group 2	Mean	Group 1	Group 2		Group 1	Group 3	Mean	Group 1	Group 3		Group 1	Group 4	Mean	Group 1	Group 4
ACT2	0.142	0.062	0.016	0.039	0.080	-0.080	0.114	0.062	0.070	0.066	0.002	-0.002	0.093	0.062	0.038	0.050	0.001	-0.001
LOS1	0.120	0.019	0.003	0.011	-0.082	0.082	0.106	0.019	0.010	0.014	0.056	-0.056	0.195	0.019	0.058	0.038	0.126	-0.126
TUA5	0.201	0.185	0.168	0.176	0.097	-0.097	0.292	0.185	0.558	0.371	0.171	-0.171	0.443	0.185	1.244	0.714	0.318	-0.318
SAND	0.072	0.044	0.002	0.023	0.018	-0.018	0.116	0.044	0.060	0.052	-0.020	0.020	0.164	0.044	0.091	0.067	0.069	-0.069
eIF4A	0.042	0.002	0.001	0.001	-0.025	0.025	0.122	0.002	0.009	0.005	0.093	-0.093	0.118	0.002	0.008	0.005	0.092	-0.092
EF1-a	0.221	0.085	0.003	0.044	0.192	-0.192	0.264	0.085	0.182	0.133	-0.191	0.191	0.195	0.085	0.479	0.282	0.020	-0.020
PP2A	0.142	0.043	0.034	0.039	-0.076	0.076	0.133	0.043	0.039	0.041	0.051	-0.051	0.081	0.043	0.016	0.030	0.010	-0.010
hnRNP	0.197	0.045	0.025	0.035	-0.151	0.151	0.177	0.045	0.113	0.079	0.081	-0.081	0.148	0.045	0.204	0.125	-0.023	0.023
18S	0.186	0.086	0.307	0.196	0.051	-0.051	0.155	0.086	0.108	0.097	-0.033	0.033	0.230	0.086	0.225	0.155	-0.100	0.100
UBQ10	0.169	0.220	0.157	0.188	-0.001	0.001	0.309	0.220	0.129	0.174	-0.264	0.264	0.509	0.220	0.609	0.414	-0.445	0.445
YLS8	0.167	0.114	0.016	0.065	-0.101	0.101	0.169	0.114	0.081	0.097	0.055	-0.055	0.204	0.114	0.173	0.143	-0.068	0.068

Table S1. Expression stability values, intra- and inter-group variances of candidate genes in groups 1 and 2, groups 1 and 3, and groups 1 and 4 analyzed by NormFinder.
